# Y-Shaped Vesica Fellea Duplex Gallbladder Causing Acute Biliary Pancreatitis

**DOI:** 10.7759/cureus.14676

**Published:** 2021-04-25

**Authors:** Eyad Gadour, Zeinab Hassan, Abdalla Hassan

**Affiliations:** 1 Gastroenterology and Hepatology, University Hospitals of Morecambe Bay National Health Service Foundation Trust, Lancaster, GBR; 2 Faculty of Medicine, The National Ribat University, Khartoum, SDN

**Keywords:** duplex gallbladder, acute pancreatitis, gall stone

## Abstract

Gallbladder duplication refers to the splitting of “gallbladder primordium” during the early embryonic development in the fifth or early sixth week. Although it is a very rare congenital abnormality and most of the patients will be asymptomatic, yet the symptomatic cases present with abdominal complaints like nausea vomiting, abdominal pain leads to cholecystitis, cholangitis, biliary colic, or pancreatitis. Herein, we present a case report of duplication of the gallbladder, which was difficult to diagnose on radiology. We report a case of a 35-year-old female who was admitted with acute gallstone pancreatitis. The diagnosis was made by magnetic resonance cholangiopancreatography (MRCP) and blood tests. She underwent an inpatient endoscopic retrograde cholangiopancreatography (ERCP) which cleared the bile duct and confirmed the diagnosis of the duplex gallbladder. The patient was then discharged home and an outpatient cholecystectomy is being planned.The duplex gallbladder may possibly be associated with other anomalies of the bile duct system. Biliary pancreatitis has been associated with such abnormality. Accurate diagnosis is crucial to achieving due to the possibility that gallbladder can be missed in imaging testing. Cholecystectomy required extreme care because these anomalies can lead to critical injuries of the bile duct and vascular system.

## Introduction

The duplex gall bladder is an exceptionally rare congenital anomaly, with an incidence of one in 4000 births and seen more commonly in females with a male/female ratio of 1:2 [1,2]. There are very few symptomatic cases reported in the scientific literature [3]. Herein, we present the first case of Y-shaped vesica fellea duplex gallbladder causing acute biliary pancreatitis.

## Case presentation

**Figure 1:**
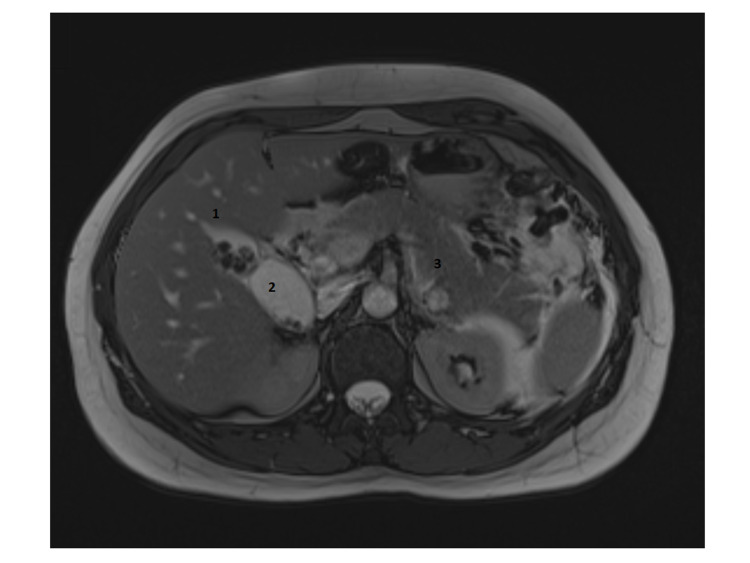
Magnetic resonance cholangiopancreatography shows duplex gallbladder (1 and 2) with acute pancreatitis (3).

**Figure 2:**
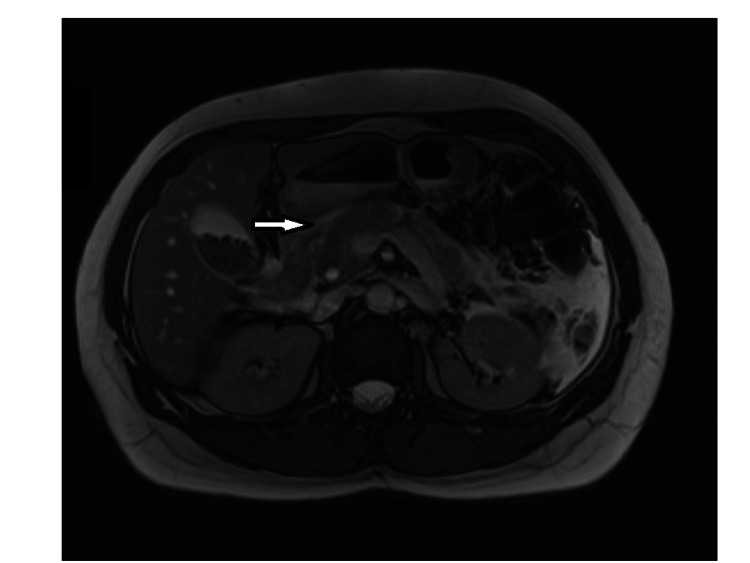
Magnetic resonance cholangiopancreatography shows Y-shaped common bile duct.

**Figure 3:**
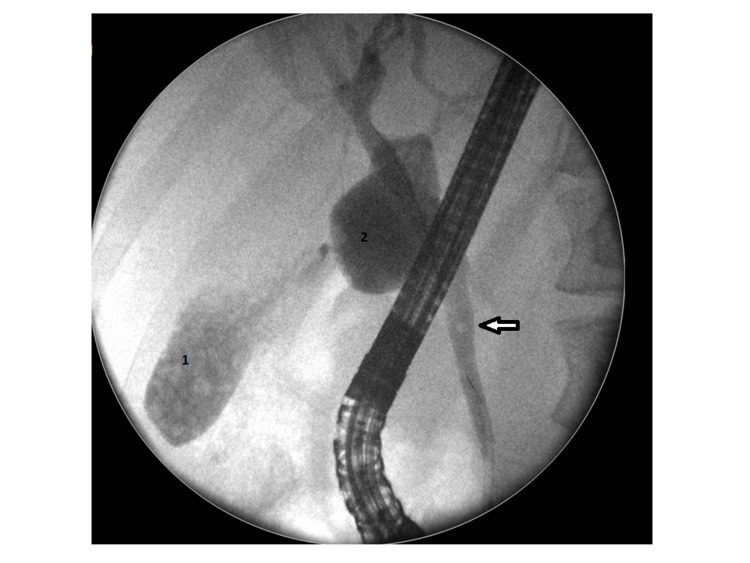
Endoscopic retrograde cholangiopancreatography shows multiple stones in both gallbladders (1 and 2) and common bile duct stones (arrowed).

**Figure 4:**
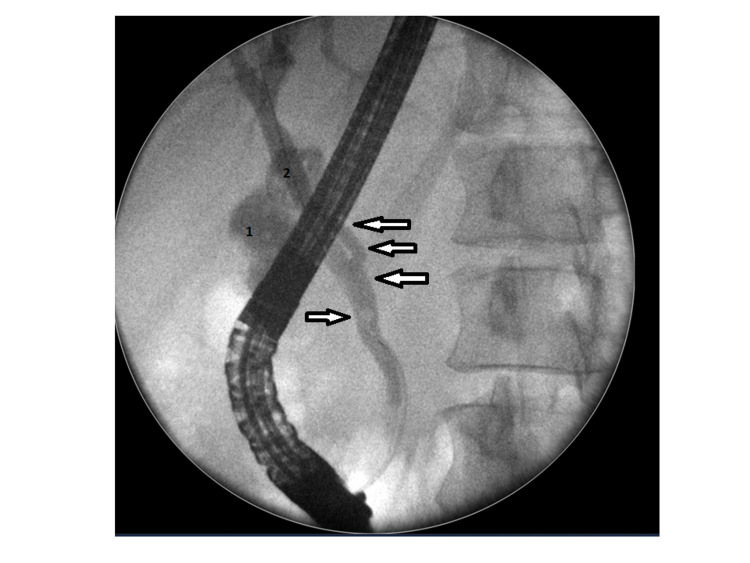
Common bile duct shows Y-shaped common bile duct with multiple stones in both gallbladders and common bile duct.

**Figure 5:**
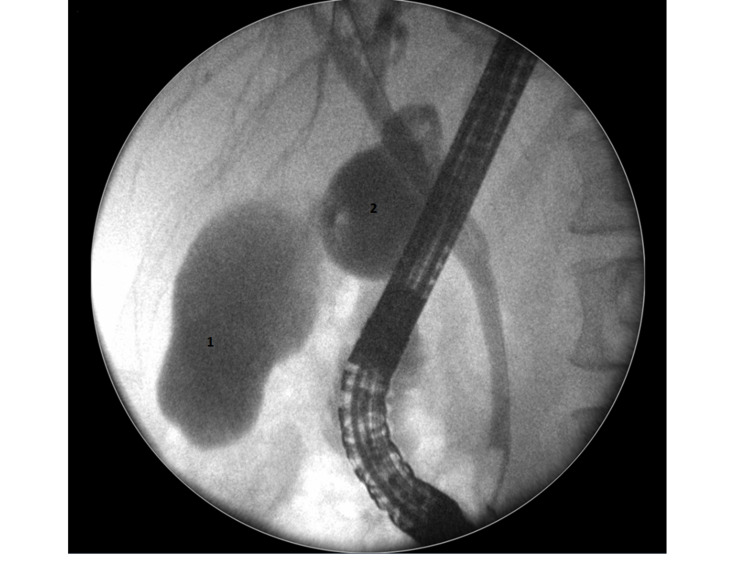
Endoscopic retrograde cholangiopancreatography cholangiogram shows complete clearance of common bile duct.

A 35-year-old normally fit and well female was presented to the acute surgical unit with severe right upper quadrant pain and tenderness for six days which was associated with nausea and vomiting. Clinically she was stable with right hypochondrial and epigastric tenderness. Her biochemical profile showed mild leucocytosis, a white cell count of 11.1, and high C-reactive protein. Her amylase level was significantly elevated to 2552 Int unit/L (normal range 22-80 Int unit/L). Conjugated bilirubin level was mildly raised at 27 mmol/L with alkaline phosphates (ALP) level at 149 U/L. Magnetic resonance cholangiopancreatography (MRCP) showed acute calicular cholecystitis in a Y-shaped vesica fellea duplex gallbladder with stones in the common bile duct (CBD) and acute pancreatitis (Figures 1 and 2). An inpatient urgent ERCP was performed within 72 hours of admission. Endoscopic retrograde cholangiopancreatography (ERCP) confirmed multiple gallbladder stones in both gallbladders as well as CBD (Figures 3 and 4). Ampullary sphincterotomy was performed and CBD stone extraction was conducted with balloon trawling. Complete clearance of CBD was achieved (Figure 5). Patient received appropriate medical treatment and safely discharged home pending an outpatient laparoscopic cholecystectomy as an outpatient.

## Discussion

Gallbladder duplication refers to the splitting of “gallbladder primordium” during the early embryonic development in the fifth or early sixth week. There are very few symptomatic cases reported in the scientific literature [3]. Different classifications have been suggested according to the differential anatomic presentation and embryological development [3].

**Figure 6:**
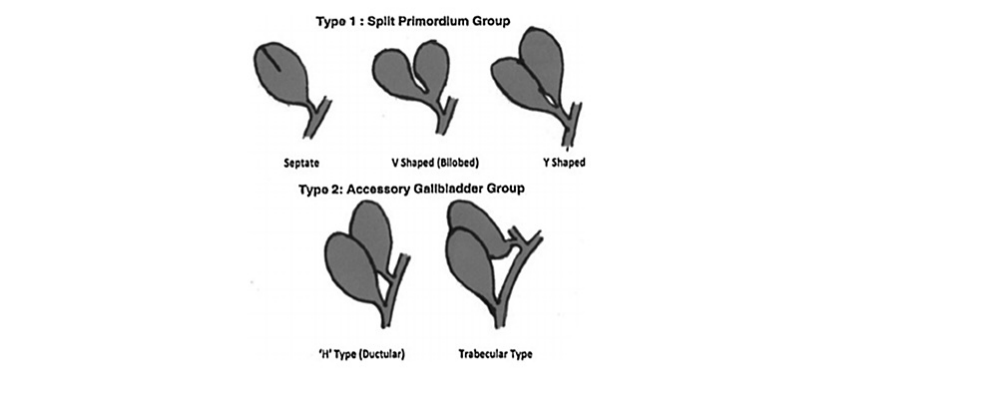
Boyden's classification. Image reproduced from Boyden [4].

According to Boyden's classification in 1929, based on 20 reported cases from 1674 to 1929 (Figure 6). The duplex gall bladder was categorized as a bilobed gallbladder with two types according to its cystic duct connection [4]. The first group categorized bi-lobed gallbladder with a single cystic duct called vesica fellea divisa and the second group includes true gallbladder duplication cases called vesica fellea duplex [2,4].

**Figure 7:**
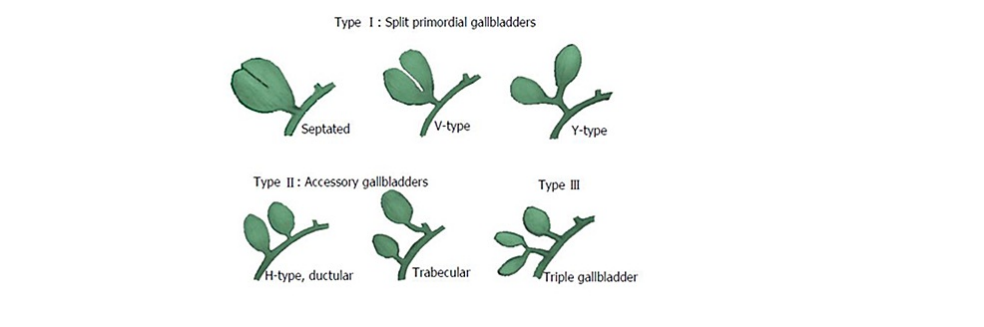
The classification of the duplex gallbladder. The image obtained from the classification by Harlaftis et al. [5].

Later, Harlaftis et al. in 1977 classified duplex gallbladder into three categories according to embryogenesis (Figure 7) [5]. Split primordial group or type 1 category includes a single cystic duct draining in the CBD. Type 1 is sub-divided into V-shape and Y-shape. Type 1-V shape cases are duplex gallbladder drain into the CBD and Y-shape anomaly includes two individual cystic ducts connected to form a single duct and drain into the CBD. The accessory gallbladder group or type 2 includes cases of more than one cystic duct drain into the CBD. These cases are also called Ductular or H type, and right and left trabecular type. Type 3 includes very rare anomalies of the duplex gallbladder which do not relevant to types A and B. The most suitable example of the type 3 group is the triple gallbladder [2,5].

Although, the duplex gallbladder is a very rare condition, yet there are around 57 reported cases so far (Table 1). People with duplex gallbladders can possibly be asymptomatic in case of not providing any hurdle in metabolic processes. The symptomatic cases present with abdominal complaints like nausea vomiting, abdominal pain leading to cholecystitis, cholangitis, biliary colic, or pancreatitis [6]. The preoperative explicit and definite diagnosis is very crucial to avoid any possible surgical surprises and complications [7]. Due to different congenital anomalies, varied anatomical presentation is associated with increased complication risk after surgical procedures like laparoscopic cholecystectomy [7]. Accurate diagnosis is crucial to achieving due to the possibility that gallbladder can be missed in imaging testing [2,8]. MRCP has better diagnostic technology than ultrasound and provided superior diagnostic details. ERCP is another radiological technology that is considered the gold standard in this case [2]. Laparoscopic cholecystectomy is the treatment choice in symptomatic cases. In the case of an incidentally identified duplicated gallbladder, prophylactic cholecystectomy is not recommended [9,10]. Some surgeons also recommend an open surgical procedure in accessory gallbladder anomalies, particularly in type 2 cases [4].

**Table 1:** Summary of the duplication of gallbladder cases reported since 1926.

Authors	Year reported	No. of cases/patient characteristics	Type of duplication
Boyden [4]	1926	20 cases	
Slaughter and Trout [11]	1933	12 cases	
Weiss [12]	1935	3 cases	
Gross [13]	1936	3-year-old male	2
Wilson [14]	1939	55-year-old female	2
Granone [15]	1984	34-year-old female	1
Udelsman and Sugarbaker [16]	1985	60-year-old female	2
Haghighi et al. [17]	2000	68-year-old female	2
Roldan-Valadez et al. [18]	2004	44-year-old male	1
Barut et al. [19]	2006	55-year-old female	1
Asbury [20]	2007	70-year-old male	1
Desolneux et al. [21]	2009	61-year-old male	1
Causey et al. [22]	2010	15-year-old female	1
Hassan et al. [23]	2012	83-year-old female	2
Shiba et al. [24]	2014	38-year-old female	1
Pillay [25]	2015	56-year-old male	1
Szczech et al. [26]	2015	26-year-old female	1
Goh et al. [6]	2015	28-year-old male	1
Gupta et al. [27]	2016	12-day-old male and 2-day-old male (two cases)	2
Rajapandian et al. [28]	2017	28-year-old male	1
Ghaderi et al. [29]	2018	38-year-old male	2
Apolo Romero et al. [30]	2018	50-year-old female	1
Boukoucha et al. [10]	2020	58-year-old female	1
Singh [1]	2021	60-year old female	1

## Conclusions

The duplex gallbladder may possibly be associated with other anomalies of the bile duct system. However, the available literature is very limited, and the ambiguity of duplex gallbladder and its association with other congenital anomalies are not clear. ERCP may be considered a confirmatory test if the radiology images are not diagnostic. No published literature yet reported this hypothesis. Cholecystectomy required extreme care because these anomalies can lead to critical injuries of the bile duct and vascular system.
